# Microvascular Decompression for Trigeminal Neuralgia Due to Venous Neurovascular Conflict: A Case Report Featuring a 2D Operative Video

**DOI:** 10.7759/cureus.112214

**Published:** 2026-07-07

**Authors:** Tiago Kiyoshi Kitabayashi Braga, Otávio da Cunha Ferreira Neto, Rodrigo Inácio Pongeluppi, Marcelo Volpon Santos, Matheus Fernando M Ballestero, Ricardo Santos de Oliveira

**Affiliations:** 1 Department of Surgery and Anatomy, Fundação de Apoio ao Ensino, Pesquisa e Assistência do Hospital das Clínicas da Faculdade de Medicina de Ribeirão Preto da Universidade de São Paulo, Ribeirão Preto, BRA

**Keywords:** microvascular decompression, microvascular decompression surgery, trigeminal nerve, trigeminal neuralgia, venous compression

## Abstract

Trigeminal neuralgia (TN) has a prevalence ranging from 0.03% to 0.3%, with an annual incidence of approximately 4.7 cases per 100,000 individuals. Among these, around 11.4% are attributed to pure venous neurovascular conflict. The transverse pontine vein emerged as the predominant offending vein. There is no consensus on the best surgical technique for these cases. However, three principal surgical techniques have been described: coagulation and cut-off, interposition, and transposition. In this report featuring an operative video, we present the case of A 37-year-old woman with shock-like and burning pain in the V2 and V3 territory on the right side for four months, associated with lacrimation and triggered by brushing her teeth or drinking cold drinks. Her pain was refractory to oxcarbazepine (2400 mg/day). Her MRI showed a neurovascular conflict on the right. She subsequently underwent microvascular decompression (MVD) surgery. During the surgery, we found a purely venous conflict. We proceeded with Teflon interposition. The patient had an uneventful recovery, with complete initial pain relief and, at three months, a favorable early outcome (Barrow Neurological Institute (BNI) pain intensity score IIIb), with pain adequately controlled on a low dose of oxcarbazepine (300 mg/day). The patient consented to the procedure and to the publication of this report. Institutional ethics committee approval was not required because of the retrospective and fully anonymized nature of the case report.

## Introduction

Trigeminal neuralgia (TN) is a paroxysmal, lancinating facial pain disorder with an estimated prevalence ranging from 0.03% to 0.3% and an annual incidence of approximately 4.7 cases per 100,000 individuals. It has a female predominance that may be related to the presence of estrogen and progesterone receptors in the trigeminal ganglion [1]. The most common underlying mechanism is neurovascular compression at the root entry zone of the trigeminal nerve, where the offending vessel is arterial in the vast majority of cases. Compression of purely venous origin is comparatively uncommon, accounting for roughly 11.4% of cases, and is therefore considered an atypical and surgically more demanding scenario [1,2].

Among the implicated veins, the transverse pontine vein has emerged as the predominant offending vessel [1]. Microvascular decompression (MVD) remains the most effective treatment for classic, medically refractory TN. However, when the conflict is purely venous, there is no clear consensus regarding the optimal surgical technique, and the postoperative outcome is more difficult to predict [2,3,4]. We present a case report featuring a two-dimensional (2D) operative video illustrating MVD with Teflon interposition for an atypical, purely venous neurovascular conflict. This report aims to illustrate the intraoperative decision-making and technical nuances of MVD in the setting of a purely venous conflict, emphasizing the rationale for interposition over coagulation and cut-off to preserve venous drainage and reduce the risk of venous infarction.

## Case presentation

A 37-year-old woman presented with a four-month history of shock-like and burning pain in the right V2 and V3 territories, associated with lacrimation and triggered by tooth brushing and cold drinks. The pain was refractory to high-dose oxcarbazepine (2,400 mg/day). Her medical history was notable only for previous bariatric surgery, with no other known comorbidities. On neurological examination, she had a Glasgow Coma Scale (GCS) score of 15, a normal motor and sensory examination, and allodynia in the right V2 and V3 territories. MRI, using post-gadolinium T1-weighted and DRIVE sequences, demonstrated a neurovascular conflict on the right side, initially interpreted as a possible arterial conflict. The purely venous nature of the conflict was recognized only intraoperatively.

Given the patient's symptomatic, medically refractory status while already on a high dose of oxcarbazepine, trigeminal nerve decompression was indicated, and MVD with Teflon interposition was selected as the most effective option. The alternatives were considered but not pursued: medical management had already been optimized; percutaneous ablation was deemed less favorable in the presence of an imaging-suspected vascular conflict; and coagulation with cut-off of the vein was avoided due to the risk of venous infarction.

The procedure was performed through a right retrosigmoid craniotomy, with the patient in the supine position, with the head fixed in a Mayfield head holder and rotated to the left. A microscope, microsurgical instruments, neuronavigation, and Teflon were used. Multimodal intraoperative neurophysiological monitoring was employed, including motor evoked potentials of the long tracts, somatosensory evoked potentials (of the median and tibial nerves), brainstem auditory evoked potentials, and direct stimulation of the cranial nerve nuclei and cranial nerves. The key surgical steps comprised a straight skin incision, retrosigmoid craniotomy, arachnoid dissection with cerebrospinal fluid drainage, identification of the trigeminal nerve, and identification of the neurovascular conflict. Only a venous conflict was found at the origin of the trigeminal root, most likely caused by the transverse pontine vein. The superior petrosal vein was protected with fibrin glue against potential intraoperative trauma. The trigeminal nerve was dissected free from the vein, and a single 3-mm piece of Teflon was interposed between the nerve and the offending vein; hemostasis was then reviewed (Video 1).

**Video 1:** Operative video

The postoperative course was uneventful. The patient spent one day in the ICU and was discharged home on postoperative day two with no neurological deficits. She experienced complete pain relief in the immediate postoperative period (Barrow Neurological Institute (BNI) pain intensity score I). At the three-month outpatient follow-up, she reported five brief pain episodes since surgery, consistent with expected postoperative discomfort rather than a recurrence of the preoperative shock-like neuralgia, with pain adequately controlled on a low dose of oxcarbazepine (300 mg/day). This corresponds to a BNI pain intensity score IIIb outcome (some pain, adequately controlled with medication).

## Discussion

Although neurovascular compression is the principal cause of classic TN, a purely venous conflict is encountered in only a minority of patients and has been recognized as a distinct, less frequent etiology since the early surgical series [1,2]. The transverse pontine vein is the most commonly implicated offending vein, as observed in the present case [1]. The atypical nature of venous compression raises specific intraoperative considerations that differ from those associated with arterial conflicts.

Three main surgical techniques have been described for the offending vein: coagulation and cut-off, interposition, and transposition, with no clear consensus on the optimal approach [2,4]. The choice of how to manage the causative vein should take into account its anatomy and drainage territory [4]. In this case, coagulation and cut-off were deliberately avoided due to the risk of venous infarction, and interposition was preferred because it relieves the conflict while preserving venous drainage [4]. Protection of the superior petrosal vein with fibrin glue further reflects the emphasis on preserving the venous architecture of the region.

A particular consideration in purely venous compression is that postoperative outcomes are less predictable than in arterial conflicts, and surgery in this subgroup has been associated with a less favorable prognosis [3]. Our patient achieved an excellent early result, with complete initial relief and good pain control at three months on a markedly reduced dose of oxcarbazepine; nevertheless, given the purely venous etiology, longer-term follow-up and counseling about the risk of recurrence remain warranted [3]. The female predominance of the condition and the reported expression of sex-hormone receptors in the trigeminal ganglion are consistent with the patient’s demographic profile [1].

This report has certain limitations. It describes a single case without a comparator group, and its conclusions are therefore illustrative rather than definitive. The three-month follow-up is relatively short and insufficient to evaluate the long-term durability of MVD for purely venous conflicts, whose outcomes are recognized as less predictable than those of arterial conflicts. Although pain outcome was graded using the BNI pain intensity scale, longer-term follow-up with serial standardized assessments would be required to confirm the durability of the result.

## Conclusions

MVD with Teflon interposition is a feasible and effective treatment for TN caused by an atypical, purely venous neurovascular conflict, providing early and substantial pain relief in this case. Preserving venous drainage by interposition rather than coagulation and cut-off appears prudent given the risk of venous infarction, and the choice of technique should be tailored to the characteristics of the offending vein. Because outcomes after surgery for purely venous compression are less predictable, continued clinical follow-up is advisable. As a single case report, these observations should be interpreted with caution and confirmed in larger series with longer follow-up.
